# Perceived driving safety and estimated blood alcohol concentration (BAC) the morning after drinking amongst young Australians attending a music festival: a cross-sectional survey

**DOI:** 10.1186/s13011-018-0157-2

**Published:** 2018-06-20

**Authors:** Mario Fernando, Johanna Buckland, Prashina Melwani, Vanessa Tent, Philip Preston, Sabrina Winona Pit

**Affiliations:** 1 School of Medicine, Western Sydney University, Building 30 Goldsmith Avenue, Campbelltown, NSW 2560 Australia; 2 STEER Project Inc, 13 Laurel Ave, Mullumbimby, NSW 2482 Australia; 3 Western Sydney University, School of Medicine, University Centre for Rural Health, School of Rural Health, University of Sydney, 61 Uralba Street, Lismore, NSW 2480 Australia

**Keywords:** Young drivers, Alcohol, Sleep, Passengers, Driving, Safety, Risk perception, Blood alcohol concentration, Morning after drinking, Health promotion

## Abstract

**Background:**

Alcohol-related motor vehicle crashes remain a significant and costly public health issue globally. Particularly young people are over-represented in these incidents. This study set out to explore the factors that influence individuals’ perceptions of their safety to drive, and the factors related to a change in intention to drive.

**Methods:**

Four hundred nine young people aged 18–40 attending an Australian multi-day music festival completed a survey measuring demographics, alcohol use, amount of sleep obtained the previous night, intention to drive, number of passengers, perceived safety to drive, estimated BAC (measured in g/210 L) and change in intention to drive following a BAC measurement via breathalysers. Statistical analyses involved univariate tests of association and multivariate logistic regression.

**Results:**

Only one in five participants felt they were completely safe to drive. Males self-rated as less safe to drive than females. Multivariate analyses showed that licence class, sleep hours, units of alcohol consumed in the past 24 h and estimated BAC had statistically significant associations with driving safety perception. Participants who slept for greater than seven hours the previous night were three times more likely to feel safe to drive than those who had less than five hours of sleep (OR 3.05 (95% CI 1.25, 7.45)). Forty-one percent of participants changed their intended time of driving after having their BAC measured with a breathalyser. There was a statistically significant association between changing the intention to drive to a later time with an increase in each extra passenger in a participant’s vehicle (OR 1.53 (95% CI 1.02, 2.30)).

**Conclusions:**

Whilst concerning behaviours relating to high-risk alcohol consumption were found, the study uncovered promising findings about young peoples’ perceptions of their safety to drive, and their propensity to change their driving intention. The strong correlation between hours of sleep, estimated BAC, units of alcohol consumed and license class with perception of driving safety suggests an increased awareness among young people and promotion of these factors may potentially improve actual driver safety. The influence of number of passengers on intention to drive later is another important consideration for future road safety research or promotion.

## Background

Drink driving is one of the leading causes of motor vehicle crashes occurring on roads within Australia [1]. In New South Wales (NSW) during 2015, alcohol was found to be a contributing factor in 47% of fatal crashes on Thursday, Friday and Saturday nights, 15% of all fatal crashes and 9% of injury crashes [1]. Whilst most fatalities involving alcohol-affected drivers occur between the hours of 12 pm to 3 am, a significant proportion occur the morning after drinking. In NSW in 2015, 12% of crashes between the hours of 3 AM and 9 AM on Saturday and Sunday mornings involved blood alcohol concentrations (BACs) over the legal limit, whereas only 7% of fatalities between these hours on weekday mornings were alcohol-affected (0.05 for fully-licensed drivers and 0.00 for provisional licence or learner drivers) [1]. This difference is augmented when considering total crashes on weekday versus weekend mornings, as 12% of crashes were alcohol-affected on weekends between 3 and 9 AM, whilst only 1.5% of crashes were alcohol affected on weekday mornings.

Currently, those most at risk of driving whilst under the influence of alcohol are young males [2]. Prior research has shown that young and less-experienced drivers are more likely to be involved in a crash when consuming alcohol [2]. Furthermore, according to the Western Australian Police Report of 2013, the highest risk group for being involved in a fatal crash were males aged 25–29, followed by males aged 17–24 [3]. In the UK in 2013, 75% of young driver fatalities were male. Although 24% of total road fatalities were over the legal BAC limit, 40% of 20–29 year-olds were over the legal BAC limit compared to only 11% of those over 40 years of age [4].

Whilst drink driving crashes have a large impact on the social and mental health of our society, they also create an economic burden. In 2006, it was found that the cost of each fatal crash was approximately $2.6 million, whilst the cost of each hospitalisation crash was approximately $266,000 [5]. Thus, by investing more into public health measures which reduce the incidence of alcohol-impaired driving the morning after drinking, there is potential for a decreased amount of resources spent on fatal and hospitalisation crashes.

Whilst alcohol consumption at outdoor music festivals in Australia is highly prevalent [6], its extent and implications on intention to drive the morning after drinking have not been thoroughly researched. A 2014 survey of music festival attendees found that 32% of participants reported “binge” drinking (6 or more drinks) on a weekly or more frequent basis in the previous 12 months, and 21% reported drinking more than 20 drinks on at least one occasion during that time [7]. High risk drinking is defined as more than 4 or 2 standard drinks daily, in men or women, respectively [8]. Therefore, this sample population represents a significantly higher rate of high-risk drinking than that of the general population, in which only 15% of men and 12% of women consume alcohol in quantities at risk or high risk to their long-term health [8].

Of particular relevance to BAC the morning after drinking is research regarding the post-absorptive state of alcohol metabolism. Traditionally, alcohol was believed to have a constant rate of clearance from the body, however physiological studies suggests that it takes longer to metabolise lower doses of alcohol due to the necessary enzymes being incompletely saturated, an effect associated with zero-order pharmacokinetics [9]. These findings imply a relatively slower BAC reduction rate at lower absolute BAC levels, such as overnight, prior to driving. Although such an effect is unlikely to impact upon full-licensed drivers the morning after moderate drinking, it is of particular applicability to younger learner or provisional drivers with 0.00 BAC limits.

Evidently, a variety of factors contribute to the social and economic burdens of alcohol-impaired driving. Two of the most vulnerable groups are youths and those driving in rural areas, both of which are highly relevant to the rural setting and young demographic of the music festival investigated by this study. Additionally, despite high rates of alcohol-related fatalities, a gap exists in the current literature regarding the rates of alcohol-impaired driving occurring the morning after drinking. One British study elicited 193 participants whose estimated and actual BACs were all over the legal limit, yet 20% believed they were still safe to drive, highlighting an incongruence between individuals’ BAC level and their perceived safety to drive [10]. A 2008 study compared binge drinkers (given 0.65 g/kg of alcohol) with non-binge drinkers (given a placebo) in their simulated driving performance, subjective ratings of intoxication and driving ability [11]. Following a binge-level consumption of alcohol, participants reported feeling less sedated and perceived their ability to drive as greater than non-binge drinkers. This finding suggests that following an acute, high dose of alcohol, the ability to assess one’s safety to drive is impaired. However, there has been a sparsity of similar research internationally that investigates the effect of acute alcohol consumption and other factors on perceived driving safety.

Another factor that may impact perceived safety to drive is sleep. A previous study found no association between participants’ number of hours of sleep and their perceived safety to drive, despite fatigue being involved in at least 16% of fatal crashes on NSW roads [1].

Because of the harms associated with driving the morning after drinking alcohol [4], research is needed to help inform public health and health promotion interventions to reduce its incidence especially for high-risk groups including young people, and those driving in country areas.

The aims of this project were to investigate the predictors of self-perceived safety to drive, and intention to drive the morning after drinking of patrons at an outdoor music festival.

## Methods

### Aim, design and setting of study

This study is a cross-sectional survey. Data was collected at a large, multi-day, outdoor music festival in rural New South Wales, Australia. The study was conducted in collaboration with a non-for-profit organisation STEER (www.steerproject.org.au). STEER regularly conducts free voluntary breath testing at major music festivals and community events with the aim of reducing the incidence of harm and injury associated with youth transport.

### Participants and materials

Participants were eligible if they were between the ages of 18 and 40 years old. Whilst the legal driving age within New South Wales is 16 years old [12], the minimum age of participants was set at 18 years old due to this being the legal drinking age within Australia [13]. In order to facilitate maximal applicability of this study to the complete music festival population of alcohol-consuming drivers, the maximum age of participants was set based on the maximum ages observed from previous research on the demographics on participants at Australian music festivals [14], as well as the highest risk age groups for alcohol-related fatalities [1, 3, 4].

Potential responders were contacted both by surveyors who roamed the campgrounds, as well as surveyors who were based at a permanent stall within the campgrounds. Participants were verbally consented to the study and provided with an information sheet outlining the purpose of the study and explaining that there would be no direct consequences of completing the survey for participants intending to drive who may be over the legal BAC limit. The breathalysers were supplied by Roads and Maritime Services NSW who managed servicing and calibration of the machines to Australian Standards. The number of individuals who declined to participate was not recorded.

### Processes, interventions and comparisons

Data collection consisted of two components. First, participants were invited to complete a survey on handheld electronic devices or paper-based surveys. The researchers screened those interested to ensure that they had consumed alcohol within the last 24 h but this did not preclude people from completing the survey. Second, after completing the survey participants were asked to estimate and record their actual BAC’s for which a handheld breathalyser was used. Participants who were intending to drive on that day were asked if knowing their BAC after having used a breathalyser changed their intention to drive. If participants answered “yes”, they were then asked if they would be driving “earlier than previously intended” or “later than previously intended”. Data collection occurred between 7 am and 12 pm each morning throughout the campsite grounds.

### Measures

The main outcome measures were: (1) perception of driving safety after consuming alcohol the night before, and (2) change in intention to drive after receiving information on actual BACs. Driving safety perception was measured against participant demographics, including age, gender and licence class, as well as the number of hours slept the previous night, number of standard drinks consumed the previous day, and self-estimated BAC. Participants were asked to determine the number of standard drinks they had consumed by using a visual guide, as attached in Appendix 1. Change in intention to drive was measured as well as the participant’s actual BAC using a breathalyser, and the number of passengers in their car.

Measures were based upon a revised version of the survey constructed by Cameron and French [10] including the number of hours the individual slept the night before, units of alcohol consumed, whether they were over or under the legal driving limit, and how safe the individual felt they were to drive. Three screening questions from AUDIT-C, a validated screening tool for alcohol consumption [15], were also included to identify those who misuse alcohol on a regular basis. Further questions were piloted to specifically target the research questions of this study. These questions included the patron’s driver’s licence status, intention to drive, passengers in the vehicle, and their estimated BAC. Estimated and actual BAC levels were categorised according to the NSW Roads and Maritime Services’ subdivision of low, medium and high range drink driving [16], i.e. below 0.049, between 0.050 to 0.079, between 0.080 to 0.149 and above 0.150. It should be noted that the unit of measurement for BAC calculated from breath testing in Australia is described in g/210 L as this represents a fixed ratio at which alcohol in the serum is expressed in expired air [17]. Drivers were also asked to identify their class of driver’s licence, in order to ascertain the driver’s level of experience and legal driving limit. Within NSW, these classes are Learners (L), Provisional 1 (P1), Provisional 2 (P2) and full licence holders, where fully licenced drivers must adhere to the legal limit of a BAC less than 0.05 and drivers holding any other licence must not have a BAC above 0.00 [16]. ‘Perceived safety’ was categorised into two categories: the “safe” group was comprised of those that reported to consider themselves “completely” or “fairly safe” to drive, whilst the “unsafe” group was comprised of those reporting they felt “completely” or “fairly unsafe” to drive. The survey used to address participants is attached in Appendix 2.

### Statistical analysis

The data were analysed using IBM SPSS Statistics (version 22). Data were assessed for normality using frequency tables, histograms and Shapiro-Wilk tests. Bivariate analysis was undertaken between the main outcome measures and each of the other variables. Categorical variables were compared using Pearson’s chi-squared tests and Fisher’s Exact tests. Continuous variables were compared using t-tests or non-parametric tests. Statistical significance was defined as *p* < 0.05. Binomial logistic regressions were used to estimate crude odds ratios and adjusted odds ratios to determine which factors are associated with a person’s perception of their safety to drive after consuming alcohol the night before.

## Results

The survey and breath test were administered to 479 participants and completed by 477; 66 participants who were drinking during the morning of the survey were excluded from the analysis.

Of the 409 participants, 60.4% were male and the median age was 21 years (Table 1). Half of the participants had a full driver’s licence. Whilst half of the participants intended to drive that day, only one in five participants felt they were completely safe to do so. Furthermore, of those intending to drive, two in five participants changed their intention of driving after reading their actual BAC level from the breathalyser (41%). Of those that changed their intentions (*n* = 85): 47% decided to leave later, 39% earlier and 14% was missing.

**Table 1:** Characteristics of study participants

Characteristics		Participants (*n* = 409)
Age, years	Median (min,max)	21 (18, 40)
	18–19 (%)	113 (27.6)
	20–21 (%)	123 (30.1)
	22–23 (%)	89 (21.8)
	24–29 (%)	71 (17.4)
	30–40 (%)	13 (3.2)
Gender	Male (%)	247 (60.4)
	Female (%)	162 (39.6)
Work Hours	Median (min, max)	30 (0, 85)
Study Hours	Median (min, max)	5 (0, 60)
Licence	No licence (%)	6 (1)
	Learner (%)	9 (2)
	Provisional driver level 1 (%)	39 (10)
	Provisional driver level 2 (%)	147 (36)
	Full licence (%)	208 (51)
Intention to Drive	Yes (%)	184 (45)
	No (%)	225 (55)
Number of passengers	Median (min, max)	2 (0, 5)
Audit-C Screening Score	Median (min, max)	7 (2, 12)
Risk of Harm from Audit-C screen	Low (%)	3 (1)
	High (%)	406 (99)
Sleep hours	Mean (SD)	5.97 (1.87)
Units of alcohol last 24 h	Median range = (min, max)	12 (0–40)
BAC Estimate	0.000 (%)	174 (43)
	0.001–0.049 (%)	149 (36)
	0.05–0.079 (%)	28 (7)
	0.08–0.149 (%)	6 (1)
	> 0.150 (%)	28 (7)
	Missing	24 (6)
Perception of safety to drive	Not safe at all (%)	37 (9)
	Fairly unsafe (%)	66 (16)
	Fairly safe (%)	224 (55)
	Completely safe (%)	82 (20)
Change of intention to drive	Yes (%)	85 (21)
	- Earlier (%)	- 33 (39)
	- Later (%)	- 40 (47)
	- Missing (%)	- 12 (14)
	No (%)	121 (30)
	Not driving (%)	201 (49)

### Perception of safety to drive

Table 2 demonstrates the relationship between self-perceived safety to drive and socio-demographics, drinking behaviour, sleep and BAC. There was a statistically significant association between the perception of safety to drive with gender, licence class, BAC estimate and actual, units of alcohol consumed in the last 24 h, hours of sleep the previous night and age.

**Table 2:** Associations between demographic, alcohol and driving factors with perceived safety to drive

Variable	All		Perceived driving safety		*P*-value^1^	χ2	dF
	*N*	*%*	*Unsafe % (n)*	*Safe % (n)*			
Gender					0.01	6.32	1
Male	247	60	30 (73)	70 (174)			
Female	162	40	19 (30)	81 (132)			
Licence Class					0.03	8.51	3
No licence/Learner	15	4	47 (7)	53 (8)			
Provisional 1	39	9	38 (15)	62 (24)			
Provisional 2	147	36	24 (35)	76 (112)			
Full licence	208	51	22 (46)	78 (162)			
Hours of Sleep					< 0.001	15.56	2
0–4	87	21	40 (35)	60 (52)			
5–7	241	59	23 (56)	77 (185)			
8–13	81	20	15 (12)	85 (69)			
Units of alcohol in last 24 h					< 0.001	18.04	3
6 or less	61	15	11 (7)	89 (54)			
6–10	135	33	19 (25)	81 (110)			
11–15	120	29	31 (37)	69 (83)			
16 or more	92	23	37 (34)	63 (58)			
Frequency of Alcoholic Drinks					0.24	1.358	1
Once a week or less	198	49	23 (45)	77 (153)			
More than once a week	209	51	28 (58)	72 (151)			
Frequency of Binge Drinking Sessions					0.07	3.15	1
Once a month or less	237	58	22 (52)	78 (185)			
Weekly or more	172	42	30 (51)	70 (121)			
Estimated BAC					< 0.001	53.94	4
0.000	174	45	10 (18)	90 (156)			
0.001–0.049	149	39	28 (41)	72 (108)			
0.05–0.079	28	7	50 (14)	50 (14)			
0.08–0.0149	6	2	83 (5)	17 (1)			
> 0.15	28	7	54 (15)	46 (13)			
Estimated BAC					< 0.001	37.98	1
0.000–0.049	323	84	18 (59)	82 (264)			
0.05+	62	16	55 (34)	45 (28)			
Actual BAC					0.07	8.67	4
0.000	314	77	26 (81)	74 (233)			
0.001–0.049	78	19	19 (15)	81 (63)			
0.05–0.079	8	2	62.5 (5)	37.5 (3)			
0.08–0.0149	5	1	40 (2)	60 (3)			
> 0.15	2	1	0 (0)	100 (3)			
	Mean	SD	Mean (SD)		*P*-value^2^	t	dF
Age (n = 409)	21.76	3.55	21.19 (3.00)	21.95 (3.70)	0.04	−2.09	407
Work hours (*n* = 408)	29.30	15.15	29.6 (15.90)	29.2 (14.91)	0.86	0.21	166.61
Study hours (*n* = 406)	9.59	11.83	9.46 (11.71)	9.64 (11.90)	0.89	−1.37	178.68

^1^ Pearson’s chi-squared test

^2^ t test, 2-tailed significance

Table 3 demonstrates the multivariate model of perceived safety based on the variables of gender, licence class, sleep hours, estimated BAC and alcoholic units consumed in the past 24 h. Gender was not associated with perceived safety. Those with a full licence reported feeling two times more likely to feel safe to drive than compared to those without (OR 2.05 (95% CI 1.20, 3.51)). In comparison to participants who had only slept for 0–4 h, those who had slept 5–7 h (OR 1.81 (95% CI 1.00, 3.28) and those who had slept for 8 h or more (OR 3.05 (95% CI 1.25, 7.45)) were more likely to feel safe to drive. Additionally, there is a trend between units of alcohol consumed and a decrease in perceived safety. Participants who had 11–15 drinks (OR 0.31(95% CI 0.11, 0.91)) or over 15 drinks (OR 0.23 (95% CI 0.08, 0.72)) were less likely to feel safe to drive than those who had consumed less than six drinks in the past 24 h.

**Table 3:** Logistic Regression Predicting Perceived Safety Based on Gender, Licence Class, Sleep Hours, BAC Estimate and Alcoholic Units Consumed in the Past 24 Hours

	Odds Ratio	Lower 95% Confidence Interval	Upper 95% Confidence Interval	*P*-value	Wald	dF
Gender^a^	0.92	0.50	1.70	0.79	0.07	1
Licence Class^b^	2.05	1.20	3.51	0.01	6.91	1
0–4 Hours of Sleep	1	–	–	–	–	–
5–7 Hours of Sleep	1.81	1.00	3.28	0.05	3.82	1
8–13 Hours of Sleep	3.05	1.25	7.45	0.02	5.96	1
Estimated BAC 0–0.049	5.05	2.72	9.39	< 0.001	26.22	1
Less than 6 Standard Drinks in 24 Hours	1	–	–	**–**	–	–
6–10 Standard Drinks in 24 Hours	0.54	0.18	1.57	0.26	1.28	1
11–15 Standard Drinks in 24 Hours	0.31	0.11	0.91	0.03	4.56	1
16 or more Standard Drinks in 24 Hours	0.23	0.07	0.72	0.01	6.38	1

^a^Males compared to females

^b^Full licensed drivers to those without a full licence

### Intention to drive after reading BAC level

Table 4 shows a statistically significant association between change in intention to drive and the number of passengers in a participant’s vehicle. This was an explorative analysis to determine if there was an association, which was found to have a moderate effect size. For each extra passenger present within a car, an individual was more likely to drive at a later time after having received their BAC reading.

**Table 4:** Associations between change in intention to drive after reading BAC levels with other variables

Continuous Variables	Mean	SD	Mean with Change in Intention to Drive (SD)		*P*-value^1^	Change to Drive Later Crude OR (95% CI)	*P*-value	t	dF
			Earlier	Later					
Passengers in car (if driving)	2.11	1.31	2.10 (1.30)	2.78 (1.25)	0.04	1.534 (1.02 to 2.30)	0.04	−2.154	60.90
Age	21.76	3.55	22.12 (4.06)	22.15 (3.01)	0.97				
Sleep hours	5.97	1.87	5.76 (1.43)	6.00 (3.39)	0.61				
Units of alcohol last 24 h	12.52	6.42	11.15 (5.52)	12.32 (6.08)	0.39				
Categorical Variables	All		Change in Intention to Drive		*P*-value^2^	χ2	dF		
	*N*	*%*	Earlier % (n)	Later % (n)					
Gender					0.12	2.45	1		
Male	247	60	39 (19)	61 (30)					
Female	162	40	58 (15)	42 (11)					
Licence Class					0.92	8.51	2		
No licence/Learner	15	4	0 (0)	0 (0)					
Provisional 1	39	9	40 (2)	60 (3)					
Provisional 2	147	36	48 (13)	52 (14)					
Full licence	208	51	44 (19)	56 (24)					
Frequency of Alcoholic Drinks					0.521	2.26	1		
Once a week or less	198	49	53 (21)	47 (19)					
More than once a week	209	51	37 (13)	63 (22)					
Frequency of Binge Drinking Sessions					0.69	0.753	1		
Once a month or less	237	58	42 (20)	58 (27)					
Weekly or more	172	42	50 (14)	50 (14)					

^1^t test, 2-tailed significance

^2^Pearson chi-squared test

## Discussion

The main findings of this study were that people with a full licence, more than four hours of sleep, estimated BAC levels of less than 0.05 and less than six alcoholic drinks in the last 24 hours felt safer to drive. Other significant findings were the high level of alcohol consumption amongst festival-goers and the increased propensity for people to change their driving intention once receiving information about their BAC.

The median number of drinks in the last 24 h for participants was 12 standard drinks, twice the number of standard drinks considered to be a binge [7]. This finding may reflect on the drinking culture present at music festivals, whereby festival-goers drink more than they would on a normal occasion.

### Perception of safety to drive

A significant association (Table 2) was found between gender and perceived safety to drive, with females being more likely to report being safe than males (81 and 70% respectively) but gender effect disappeared in the final multivariate model (Table 3). This difference may be attributable to the wider community’s perceptions about females being less involved in alcohol-related crashes than males [18], as well as gender differences in alcohol consumption [19] and high-risk behavior being more common among males. In the context of driving the morning after drinking, males may evaluate themselves as being less safe to drive as a consequence of relatively higher levels of alcohol consumption and higher risk behavior amongst males.

### BAC estimate

Individuals with a higher estimated higher BAC felt significantly less safe to drive relative to those who had estimated a lower BAC. Despite this, 45% of participants who estimated a BAC over the legal limit still felt safe to drive. This finding is similar to other research conducted in the UK which found that 20% of participants who thought they would be over the legal limit, still considered themselves safe to drive [10]. This may represent the increased confidence and loss of inhibition towards driving safety that occurs in individuals consuming significant amounts of alcohol [11]. These results indicate that overall, estimated BAC was a valuable tool in determining the safety to drive of our participants. However, it does not appear to be an absolute determinant for perception of safety to drive. The role of alternate factors including sleep may also contribute to these findings.

### Sleep

In recent years a sustained effort has been made by the regulators of road safety in Australia to increase awareness around factors affecting driving quality and safety other than alcohol consumption [20]. A study conducted by the Research of Accident and Road Safety, found that participants perceived drink driving to have a greater impact upon driving quality and safety when compared to sleep deprivation, even though significant sleep deprivation has been found to have effects on driving safety comparable to alcohol consumption [21]. Overall, our study showed that a greater number of hours slept after drinking was associated with a perception of increased safety to drive. This accords with the awareness of the importance of sleep for driving safety and the positive effect of road safety campaigns.

### Alcohol over the last 24 h

Generally, participants who had consumed more than six units of alcohol over the preceding 24 h were more likely to rate themselves as fairly unsafe or unsafe to drive the morning after drinking. However, the strength of this association in the multivariate model was not as strong as that of the association between BAC estimations and safety to drive, with the exception of those that drank more than 16 drinks in the last 24 h. This finding represents the notion that the number of standard drinks an individual consumes before feeling unsafe to drive may vary considerably based upon individual factors such as gender and body mass index. By estimating BAC, an individual is able to consider their own levels of intoxication the evening before, potentially by subconsciously taking into account these individual factors along with the number of standard drinks consumed.

### Change in intention to drive with number of passengers

There was a statistically significant association (Table 4) between the number of passengers in a driver’s car and their change in intention to drive after obtaining a BAC measurement. A higher number of passengers in a participant’s car was correlated with an increased likelihood of a participant changing their intention to drive. This was a finding of an explorative analysis and may have been a finding due to chance. The finding may be attributed to drivers feeling personally responsible for the safety of their passengers, and thus leaving later, or pressure from their passengers to leave at an earlier time if their BAC was under the legal limit. Although a result of an exploratory analysis, this is valuable information for encouraging event holders and venues to have BAC measuring tools available, to result in more aware drivers who can make more informed decisions about when is safe to drive. These tools may be particularly useful in public, visible areas, where passengers themselves can see their driver’s BAC level and arrange alternate transport if necessary. It may also guide public health measures to target individuals sense of responsibility towards their passengers. Whilst a multitude of studies have been previously conducted to determine passenger influences on risky driving behaviour in young adults, there is limited information regarding the relationship between the number of passengers and the perceived safety of drivers. Investigating this relationship could be a potential area for future research.

### Limitations

Study limitations included the inability to determine causal inference, lack of a population based-study sample and self-report. As the survey involved a breath-testing component, participants who were more cautious and thus more willing to find out their BAC may have been more likely to complete the survey. There is some selection bias in the fact that participants who were mindful of the number of drinks they had consumed, and diligent enough to answer all of the survey questions, may have been inherently more attuned to driver safety and less likely to drive the morning after drinking. Additionally, the number of individuals who declined to participate was not recorded which is a study limitation. Furthermore, learners and provisional licence holders are legally required to have a blood alcohol concentration of 0.00 in Australia, and it is likely that they may be more likely to participate due to the zero-tolerance of the legal alcohol limit for these driver classes.

Additionally, the survey administered did not include a question regarding concurrent illicit drug use during the festival. A previous study reported a three times higher rate of illicit drug use in young Australian music festival attendees than the average young Australian population [14]. Although this study did not report use of illicit drugs itself, there is a possibility that drug use during the festival may have contributed to an individual’s perception of being unsafe to drive.

Another limitation was that some participants had positive blood alcohol readings at the time of being surveyed. It is possible that this could have influenced a participant’s ability to complete the survey correctly. There was also the potential for inaccuracy from the breath testing equipment, which was minimised by ensuring all equipment was maintained and calibrated according to Australian standards.

Furthermore, participants were only asked, a) if they would drive at the same time, b) earlier or, c) later after receiving their blood alcohol concentration, with some individuals expressing that they would instead have another passenger drive. These responses were not accounted for in the results, but do nonetheless constitute a positive safe driving behaviour change the morning after drinking.

The study was not designed to accurately assess the impact of a BAC reading as a behavioural intervention. It was interesting to observe the reactions of participants towards their BAC results, and the consequent actions they made in response, for example waiting to “sober up” or changing drivers. Many were surprised by their result (whether lower or higher than expected), and used this information to make decisions regarding their travel plans. The thought processes behind these actions would be an area of research worthwhile pursuing. Future studies could more aptly elucidate the effect of BAC readings on an individual’s change in intention to drive.

Forty-eight percent of participants surveyed were learners and provisional licence holders, compared to 13.5% of licence holders registered within NSW as of June 2017 [22]. This further emphasises the disparity between the general population and the population sampled throughout this paper. Additionally, this research may not be representative of all Australian youth due to the specific population that attends music festivals. However, the findings may potentially be generalisable to young festival populations. This is important because there are indications that music festival goers may have higher rates of ‘risky single occasions of drinking’ than their peers of the same age, and thus may be at higher risks of driving crashes with alcohol involvement [23].

### Implications

Overall, this study has implications for reducing the incidence of driving the morning after drinking whilst over the legal BAC limit. Encouraging young individuals to self-evaluate their BAC prior to driving has the potential to increase self-awareness regarding safety to drive. Further interventions targeting youth regarding how BACs are measured, the lasting effects of alcohol within the body hours after drinking cessation has occurred, and education on the effects of compounding factors resulting in decreased safety on the road such as sleep may support this endeavor.

Only one in five participants that intended to drive felt they were completely safe to drive. This indicates that more awareness may need to be placed on safe driving practices, or alternative transport options by music festival organisers for those participants who do not feel safe to drive.

On an observational note, many individuals encountered during the data collection phase of this study were unsure as to how to estimate their BAC as they had never been breath-tested before. Continuing to encourage the use of free voluntary breath-testing services at licensed venues and festivals could assist young people in achieving greater awareness of individual differences in BAC both at the time of drinking and after drinking cessation.

Forty-one percent of participants changed their intention of driving after having their BAC measured with a breathalyser, with an association between a change in intention to drive and the number of passengers present within the car. By having increased access to breath-testing facilities, individuals are made better aware of their level of impairment, and thus able to make more informed decisions on their driving behaviours to the benefit of themselves and their passengers. The provision of voluntary breath testing services to delay patrons from driving when over the legal limit is also a potential area for future research.

## Conclusions

This study found that young people attending a music festival showed concerning levels of high-risk drinking behaviours, with large amounts of alcohol consumed in a 24-h period. This behaviour poses a risk to those intending to drive and other road users. Promisingly however, the young people involved in this survey showed a strong correlation between their self-estimated BAC, the number of hours they had slept and amount of alcohol consumed, and their perception of safety to drive. This suggests that there is a consciousness of the factors related to driving safety. The survey participants also appeared to consider their safety to drive as important, with a significant number choosing to drive later than intended following a BAC measurement, especially if they were responsible for carrying passengers.

These findings present important implications for alcohol and road safety public health messages and interventions directed towards young people.

## Figures and Tables

**Fig. 1:**
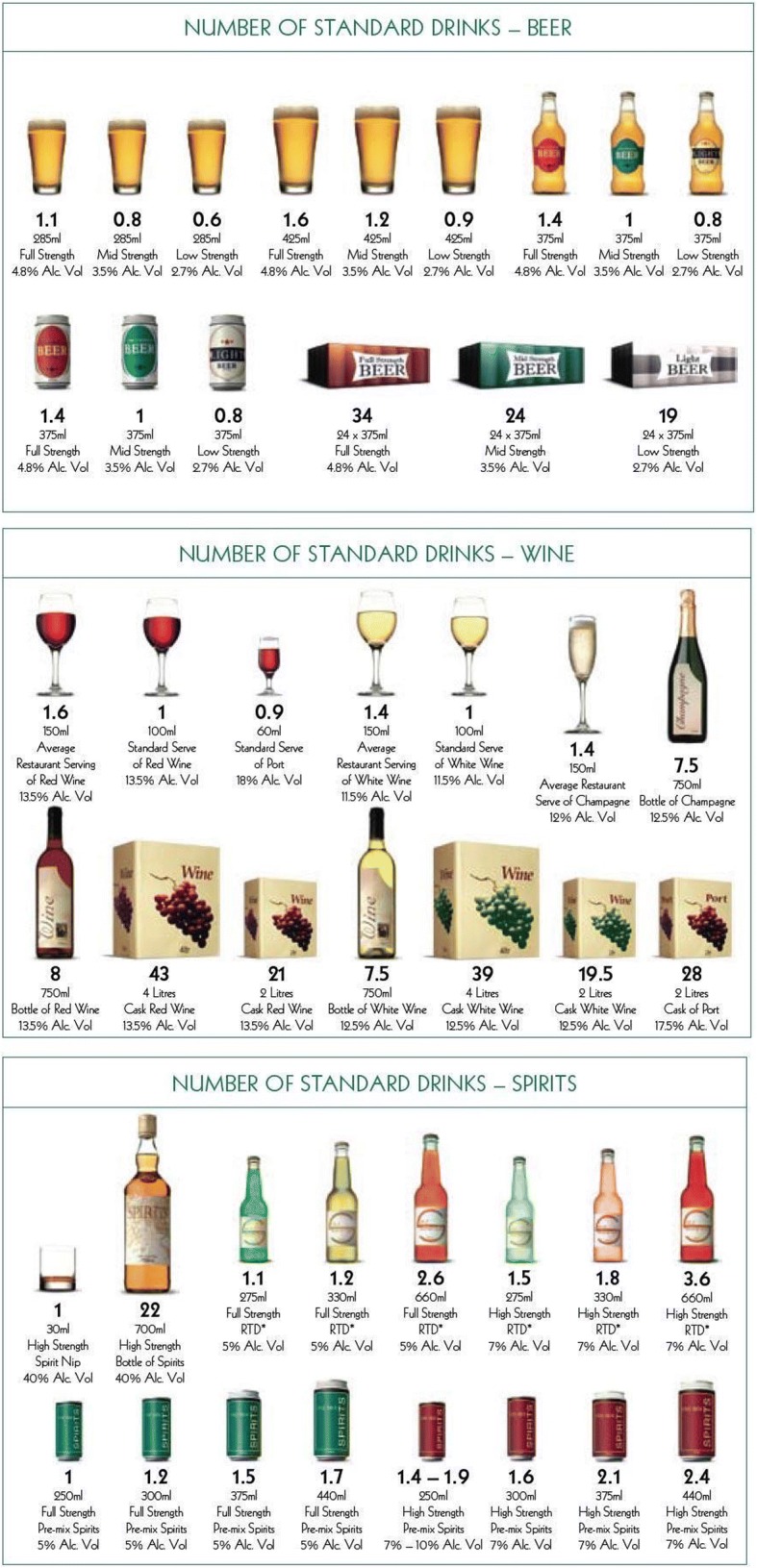
Australian National Health and Medicine Research Council standard drinks guide

**Fig. 2:**
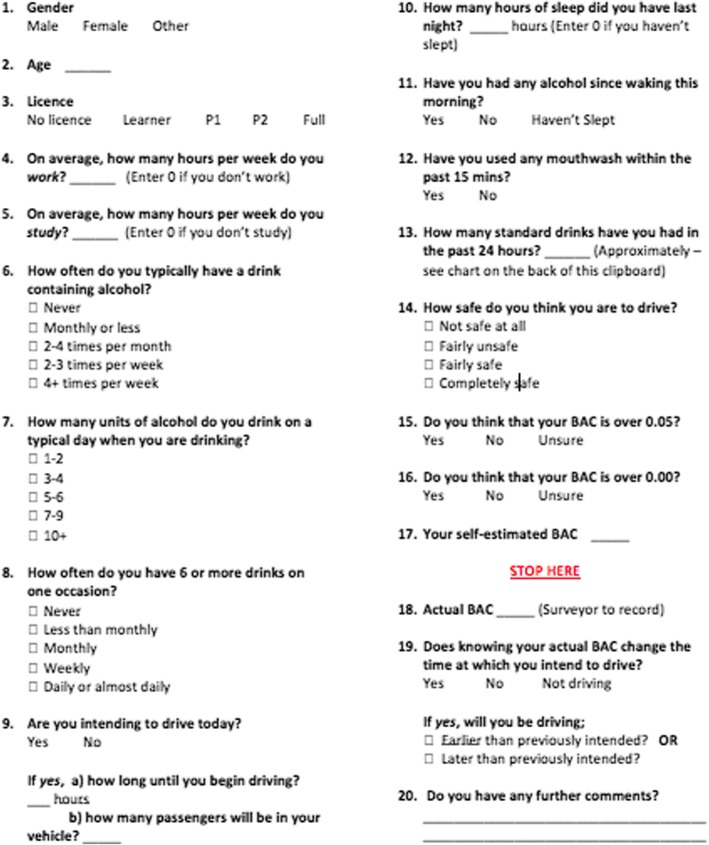
Survey tool

## Appendix 1

### Standard Drinks Guide

As published by the Australian National Health and Medicine Research Council [24]:

## Appendix 2

### Survey

Thank you for giving your time. Please only complete this survey if you are aged 17 and over. By completing this survey you acknowledge that you have read and understood the Participant Information Sheet and are giving your consent to being included in the study. You may withdraw at any time until the survey is submitted. In this survey blood alcohol concentration will be referred to as BAC.

Thank you for your time. Please place the survey in the box. If completion of this survey has caused any distress or if you have concerns about your drinking habits, please consider the following options: Contact your own GP or Helpline on: 13 11 14 or Visit http://www.beyondblue.org.au to identify other sources of help. This study is being carried out under the 2016 community research program, Western Sydney University (HREC Approval Code H11327.

## Abbreviations

BAC: Blood Alcohol Concentration (measured in g/210 L)
NSW: New South Wales
